# Effects of a Mat Pilates Exercise Program Associated with Photobiomodulation Therapy in Patients with Chronic Nonspecific Low Back Pain: A Randomized, Double-Blind, Sham-Controlled Trial

**DOI:** 10.3390/healthcare12141416

**Published:** 2024-07-16

**Authors:** Jeanne Karlette Merlo, Adriano Valmozino da Silva, Juliano Casonatto, Alex Silva Ribeiro, Eros de Oliveira Junior, Ana Paula do Nascimento, Raphael Gonçalves de Oliveira, Cosme Franklim Buzzachera, Rubens Alexandre da Silva, Andreo Fernando Aguiar

**Affiliations:** 1 Postgraduate Program in Rehabilitation Sciences, Northern University of Paraná (UNOPAR), Londrina 86041-120, PR, Brazil; j_fisioedu@hotmail.com (J.K.M.); adriano.valmozino@gmail.com (A.V.d.S.); juliano2608@hotmail.com (J.C.); erosjunior@hotmail.com (E.d.O.J.) anaapaulanascimento@gmail.com (A.P.d.N.); rgoliveira@uenp.edu.br (R.G.d.O.); 2 Postgraduate Program in Physical Exercise in Health Promotion, Northern University of Paraná (UNOPAR), Londrina 86041-120, PR, Brazil; alex-silvaribeiro@hotmail.com; 3 Postgraduate Program in Human Movement Sciences, State University of Northern Paraná (UENP), Jacarezinho 86400-000, PR, Brazil; 4 Department of Public Health, Experimental Medicine and Forensic Science, University of Pavia, 27100 Pavia, Italy; cosme.buzzachera@unipv.it; 5 Program de Physiothérapie de L’université McGill Offert en Extension à L’UNIVERSITÉ du Québec à Chicoutimi (UQAC), Québec, QC G7H 5B8, Canada

**Keywords:** disability, exercise, laser, light-emitting diodes, lumbar spine, rehabilitation

## Abstract

Objective: To investigate the effects of combining a Pilates program with photobiomodulation therapy (PBMT) in patients with chronic nonspecific low back pain (CNLBP). Methods: Thirty-eight adults with CNLBP were randomly assigned to two groups: Pilates exercise + active PBMT (PIL + PBMT) or Pilates exercise + sham PBMT (PIL + SHAM). Both groups performed an 8-week mat Pilates program and received PBMT on their lumbar muscles 10 min before and after each session. The following variables were assessed before and after intervention: peak pain intensity, postural balance (i.e., center of the pressure [A-COP], velocity anteroposterior [Vel AP], and velocity mediolateral [Vel ML]), perceived disability (i.e., Oswestry Disability Index [ODI] and Roland Morris Disability Questionnaire [RMDQ]), and pain-related fear of movement (i.e., Tampa Scale of Kinesiophobia [TSK], Fear Avoidance Beliefs Questionnaire [FABQ], and Pain Catastrophizing Scale [PCS]). Results: Postural balance variables showed no statistically significant differences (*p* > 0.05) across time or between groups. The groups showed similar (*p* < 0.05) reductions in peak pain intensity, ODI, RMDQ, and PCS scores, but no statistically significant difference (*p* > 0.05) in TSK and FABQ scores. Conclusion: The mat Pilates program reduced peak pain intensity, perceived disability, and pain catastrophizing in adults with CNLBP, but PBMT had no additional effect on these variables. Mat Pilates alone or combined with PBMT was not able to improve postural balance.

## 1. Introduction

Low back pain (LBP) is the most common musculoskeletal condition globally, accounting for the majority of years lived with disability (YLDs) [1]. LBP affected 619 million people worldwide in 2020 and is expected to affect over 800 million people by 2050 [1]. The prevalence of LBP increases gradually with age, reaching 80–89 year [2], and is associated with a higher absenteeism rate in physically demanding jobs [3]. In the United States, 15.4% of the workforce reports approximately 10.5 lost workdays each year due to LBP, equating to roughly 264 million [1]. Therefore, LBP is a serious public health concern that reduces productivity and presence at work, resulting in a significant economic cost to people, healthcare providers, and societies [1,4].

LBP is defined as “pain, muscle tension, or stiffness localized below the costal margin and the inferior gluteal folds, with or without lower extremity pain” [5,6] and can be classified into two main categories: (a) specific LBP (pain caused by a particular disease or structural problem in the spine—e.g., infection, tumor, fracture, inflammatory process, or radicular syndrome) and (b) nonspecific LBP (pain not attributable to a known cause) [4,6]. Nonspecific LBP accounts for 85–90% of all cases [7], and can be classified as chronic nonspecific LBP (CNLBP) when symptoms last more than 12 weeks [6]. Chronic LBP has been associated with decreased postural control, reduced proprioceptive sensibility, functional impairment, pain-related fear of movement (kinesiophobia), and depression [8,9,10,11,12,13,14]. Therefore, developing effective strategies for treating chronic LBP should be prioritized in public health.

Exercise therapy has been identified as an essential nonpharmacological therapeutic strategy for patients with chronic LBP [15]. A recent meta-analysis study [16] of 249 exercise treatment trials indicated a clinically significant moderate effect (mean difference [MD] of −15.2) in favor of exercise therapy compared with no treatment, usual care, or placebo for pain outcomes in patients with chronic LBP. Pilates exercise therapy was found to be one of the most effective exercise therapies for adults with CNLBP, with standardized MDs of −1.86 and −0.92 for pain and disability outcomes, respectively, when compared with no treatment [15]. Pilates is a mind–body exercise that improves static and dynamic stability, strength, core stability, flexibility, muscle control, postural control, and breathing [17]. It has also been shown to reduce kinesiophobia in patients with CNLBP compared with minimal intervention or no treatment [18]. Furthermore, a recent network meta-analysis [19] of 118 trials (9710 participants) found that Pilates exercise was more likely to reduce pain (93%) and disability (98%) in adults with chronic LBP than other exercise modalities (i.e., mind and body, core-based, strength, combined, stretching, McKenzie, and aerobic). However, the Pilates studies had a low-to-moderate level of evidence quality, indicating that further high-quality research is needed to demonstrate the effectiveness of this intervention in the treatment of CNLBP [15,18,19].

Manual therapy, acupuncture, cryotherapy, ultrasound, thermotherapy, traction, transcutaneous electrical nerve stimulation, and photobiomodulation therapy (PBMT) are complementary strategies to physical exercise that are commonly used in clinical settings. The latter has attracted widespread attention from the clinical community and has been thoroughly investigated in the literature, although its effectiveness in the treatment of CNLBP remains uncertain. PBMT is the use of light to stimulate tissue regeneration, reduce inflammation, and provide analgesia, often with a low-power light source (laser or light-emitting diodes [LEDs]) [20]. PBMT works by interacting between irradiated light and photoreceptors (e.g., cytochrome c-oxidase) in mitochondria, resulting in increased energy production (i.e., adenosine triphosphate [ATP]) [20,21,22]. Furthermore, PBMT has been shown to promote analgesic effects by modulating inflammatory mediator levels [23], such as prostaglandin E_2_ [24], and improving muscle regeneration by promoting satellite cell expression/activity [22]. A recent meta-analysis study [25] of 12 randomized controlled trials (pooled *n* = 1046) indicated that PBMT had a small, nonsignificant effect on pain intensity (MD = −3.95) and disability (MD = −4.83) in patients with CNLBP. Combining PBMT with physical exercise (i.e., aquatic and multimodal) resulted in a greater (but not significant) effect on pain intensity (MD = −11.7) and disability (MD = −5.26) compared with exercise alone. This suggests that exercise + PBMT may maximize CNLBP treatment effects. Importantly, only three studies compared exercise + PBMT with exercise alone and presented low quality evidence [25], and no studies have yet investigated the combined effects of Pilates and PBMT in the treatment of CNLBP. Given that Pilates exercise is thought to be more effective than other exercise modalities for treating chronic LBP [19], the combination of Pilates and PBMT may result in better treatment effectiveness.

This study aimed to investigate the effects of combining a mat Pilates exercise program with PBMT for the treatment of CNLBP. Given the supposed beneficial effects of Pilates [15,18,19] and PBMT [24,26,27], we hypothesized that the mat Pilates program combined with PBMT would outperform the mat Pilates program alone in improving postural balance and decreasing pain intensity, perceived disability, and pain-related fear of movement in patients with CNLBP.

## 2. Materials and Methods

### 2.1. Participants

Adults with CNLBP were recruited using social media advertisements, hospitals, physiotherapy clinics, and the local community. To be eligible, individuals are required to fulfill the following criteria: (a) age 18–64 year, (b) self-reported CNLBP (≥3 days per·week) for at least 3 months, and (c) no involvement in a supervised exercise program (>2 days per·week) over the past 6 months. Participants were excluded if they (a) had medical comorbidities (e.g., cardiovascular, neurological, endocrine, or metabolic diseases) or physical limitations (e.g., orthopedic or rheumatic diseases, muscular injury, fibromyalgia, or pain) that could affect their ability to perform the Pilates exercises; (b) had previous back and lower limb surgery; (c) had any specific spinal disease (e.g., spinal osteoporosis, cancer, herniated discs, facet syndrome, or spinal stenosis); (d) were taking any medication that could affect their somatic and cognitive functions (e.g., muscle relaxants, antidepressants, sleeping pills, allergy medicines, or stimulants); and (e) had any skin diseases (e.g., erysipelas, eczema, dermatitis, psoriasis, or urticaria) in the lumbar region that might be aggravated by PBMT irradiation. All participants were informed of the study’s protocols, associated risks, and benefits and provided signed informed consent. The study protocol was reviewed and approved by the research ethics committee of the Northern University of Paraná (protocol: 4.669.408) and registered at clinicaltrials.gov (NCT04887987). All study protocols followed the ethical standards outlined in the Helsinki Declaration.

### 2.2. Experimental Design

A randomized, double-blind, sham-controlled trial was conducted to determine the effects of a mat Pilates exercise program combined with PBMT on pain intensity, postural balance, perceived disability, and pain-related fear in adults with CNLBP. Following baseline measurements, participants were randomly assigned to one of two groups (https://www.random.org): Pilates exercise + active PBMT (PIL + PBMT) or Pilates exercise + sham PBMT (PIL + SHAM). The baseline characteristics of the participants from both groups are shown in Table 1. The groups followed the same mat Pilates exercise program for 8 weeks (2 days per·week) and received either active or sham PBMT (10 min before and after each exercise session, totaling 20 min).

**Figure 1:**
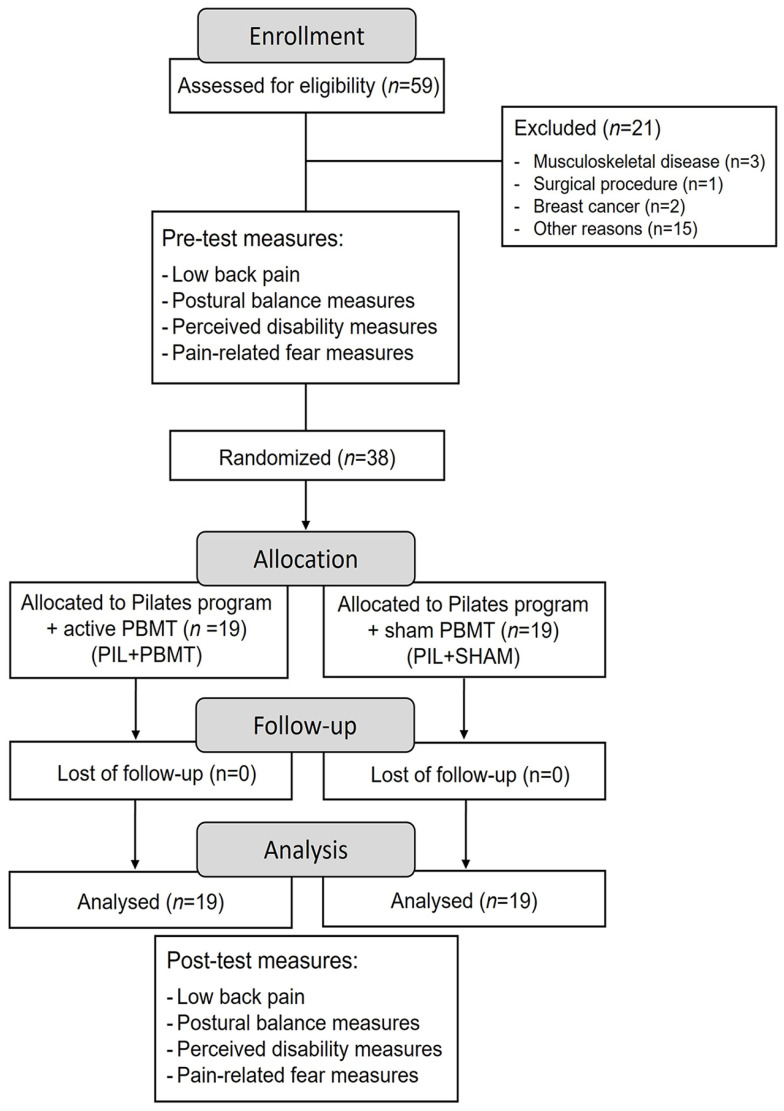
CONSORT flowchart.

The following dependent variables were measured before and after the intervention period: mean and peak LBP, postural balance, perceived disability (i.e., Oswestry Disability Index [ODI] and Roland Morris Disability Questionnaire [RMDQ]), and pain-related fear (i.e., Tampa Scale of Kinesiophobia [TSK], Fear Avoidance Beliefs Questionnaire [FABQ], and Pain Catastrophizing Scale [PCS]). The ODI, PCS, and TSK can be used without permission by students, physicians, clinical practitioners, and non-funded academic users [28], whereas the RMDQ can be used without consent in research and clinical practice [29]. A CONSORT flowchart is shown in Figure 1.

### 2.3. Sample Size Calculation

The sample size was computed using the G*Power software (version 3.0.1; Dusseldorf, Germany) with the following parameters: repeated measures analysis of variance (ANOVA), between-factors, effects size (ES) = 0.52, α = 0.05, *β* = 0.80, number of groups = 2, and number of measurements = 2. Cohen’s ES was calculated using the between-group difference (Pilates vs. minimal intervention) in pain and disability outcomes among patients with chronic LBP [30]. Pain and disability were considered for the sample size calculation because they are significantly associated with pain catastrophizing in patients with chronic LBP [31,32,33]. To reject the null hypothesis with an 80% likelihood (actual power), at least 32 participants (*n* = 16 for each group) were required. Each treatment group had 19 participants to increase statistical power.

### 2.4. Interventions

#### 2.4.1. Mat Pilates Exercise Program

**Figure 2:**
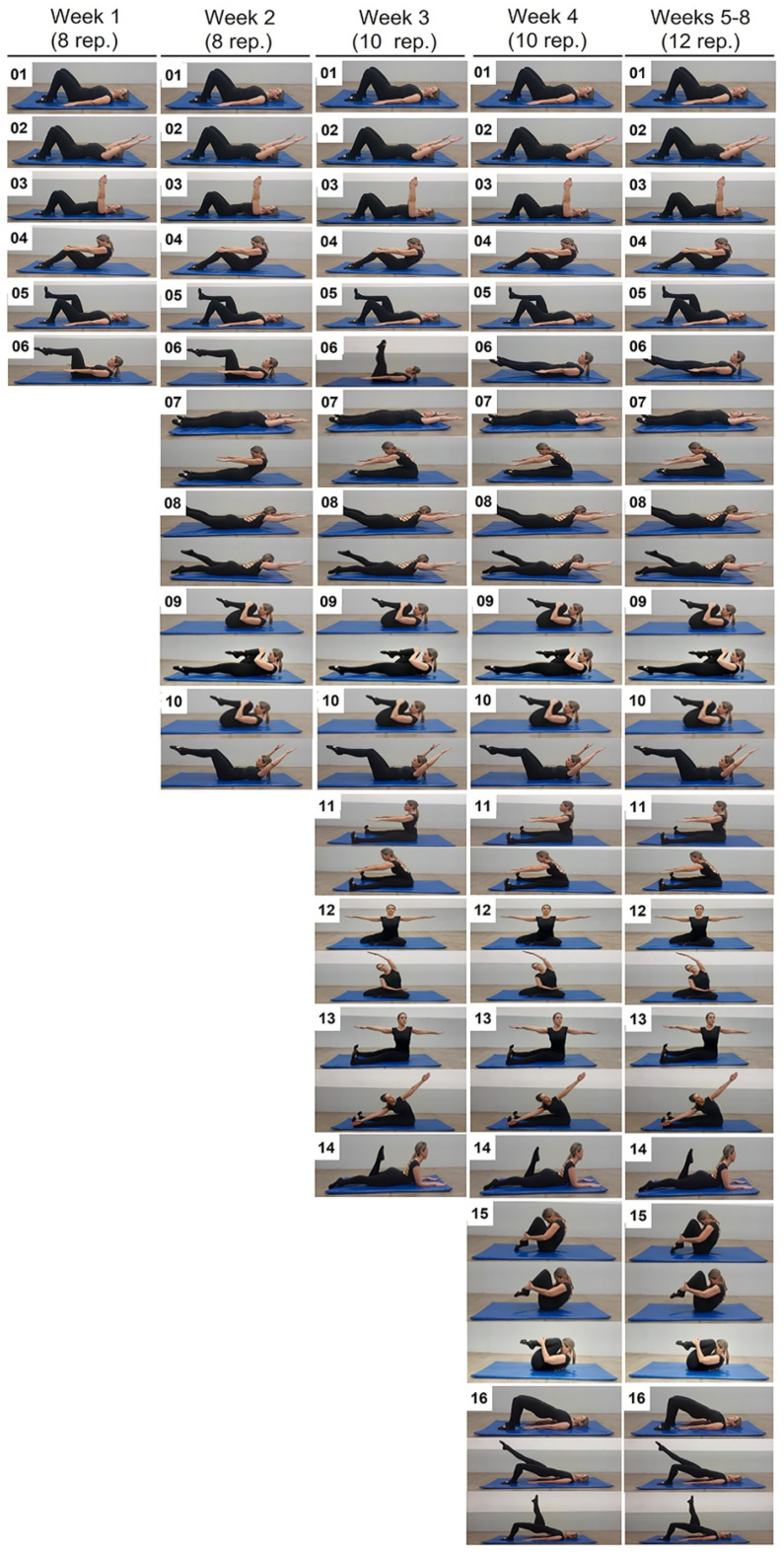
Mat Pilates program.

Both groups (PIL + PBMT and PIL + SHAM) completed the same mat Pilates exercise program for 8 weeks (2 days per·week; Figure 2). The Pilates program was based on previous studies [19,34,35,36,37] and clinical experience from a physiotherapist certified in the Pilates method (with over 13 years of experience) who supervised all exercise sessions. The Pilates program also progressed in terms of the number of exercises (6–16), repetitions (8–12), and level of exercise complexity (basic, intermediate, and advanced) (for details on exercise protocol, see Supplementary Table S1). It primarily aimed to increase the stability and mobility of the lumbar spine. The program was adapted individually, with progression criteria including the participant’s ability to engage specific muscles, the reduction of postural compensations, the acquisition of a well-controlled movement, and a more stable posture during the exercises [38]. Participants were instructed to breathe correctly during the exercises (i.e., inhale through the nose, expand the ribs laterally, and exhale through the mouth, closing the rib cage down and in). Sessions were performed in the afternoon (between 2 and 4 p.m.) and lasted around 50 min, starting with a 10 min awareness of powerhouse activation, shoulder girdle, and pelvic girdle, while also focusing on breathing. Throughout the intervention, no participant reported any adverse effects.

#### 2.4.2. Photobiomodulation and Blinding Procedures

**Figure 3:**
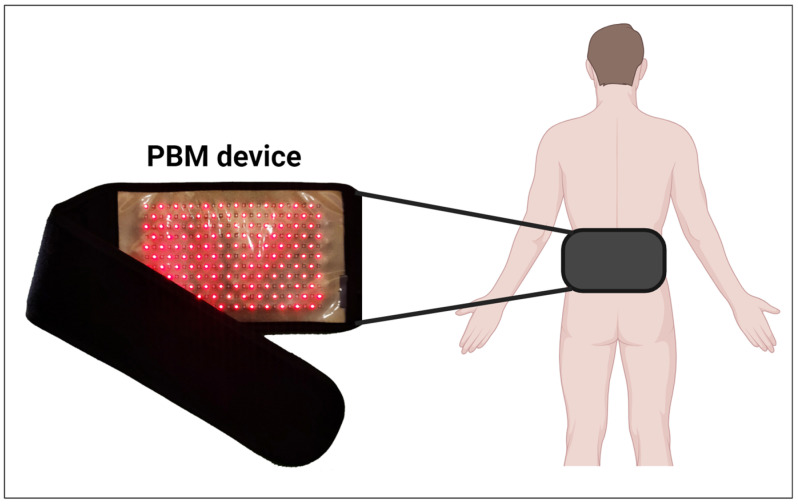
Photobiomodulation (PBMT) device and its application on the lumbar region.

PBMT was performed on the lumbar spine between L1 and L5, using an LED device (Sportllux^®^, Cosmedical, São Paulo, Brazil) with 264 LEDs (132 red and 132 infrared; Figure 3). The irradiation parameters are shown in Table 2. The dosimetry was based on a previous study of PBMT in patients with CNLBP [39]. Both groups (PIL + PBMT and PIL + SHAM) received the same treatment time (10 min before and after exercise sessions, for a total of 20 min), with the device switched off in the PIL + SHAM group. Participants were prone and wore a dark blindfold over their eyes during PBMT procedures; therefore, they were blind to the PBMT parameters. The physiotherapist and evaluator who administered Pilates exercises and questionnaires, respectively, were blinded to the PBMT treatments (PIL + PBMT and PIL + SHAM). The researcher responsible for the statistical analysis was also blinded to the treatments.

### 2.5. Outcomes Measures

#### 2.5.1. Low Back Pain

Pain severity was the primary outcome, and it was self-reported using a 10-point numerical rating scale (NRS), with extremities designated as 0 (no pain) to 10 (unbearable pain). were instructed to draw a vertical line at a scale point that best matched their level of pain throughout the day. Pain intensity was recorded at night (between 7 and 8 p.m.) for the first 3 days before the intervention (basal) and the last 3 days after the intervention. Peak pain intensity was defined as the highest value reported over the 3 days before and after intervention. We chose a daily pain report (rather than movement-specific acute pain measures like getting out of a chair or climbing stairs) because LBP often has an oscillatory pattern of timing and intensity throughout the day, depending on each patient’s activities of daily living and physical condition. Therefore, an analysis of daily global LBP can provide a more accurate diagnosis of the patient’s LBP condition. NRS has been characterized as a reliable method for determining LBP severity [40].

#### 2.5.2. Postural Balance

Postural balance was the primary outcome assessed using a force platform (BIOMEC400-412, EMG System do Brazil Ltda, São Paulo, Brazil). All participants performed three attempts at static unipedal standing postures (right and left legs) for 30 s each, with a 1 min rest interval in between. The mean of three attempts was used for analysis. Participants were instructed to stand on a platform (barefoot), arms parallel to the trunk, eyes open, and look at a target (circle) on a wall 2.5 m away. The vertical ground reaction force measurements from the force platform were captured at 100 Hz. All force signals were filtered by a 35 Hz low-pass filter (Butterworth filter). The signals from the four force platform sensors were transformed into center of pressure (COP) data using computerized stabilography, which was then assembled with MATLAB procedures (The Mathworks, Natick, MA, USA). Stabilographic analysis of the COP was used to derive the following balance parameters: area of the COP (A-COP), velocity anteroposterior (Vel AP), and velocity mediolateral (Vel ML). There is an inverse relationship between these variables and postural balance, with lower values indicating better balance. We opted to examine postural balance because previous studies found increased postural instability in patients with CNLBP [41,42,43], and exercise appears to be a countermeasure for this condition [44]. COP parameters are a reliable tool for measuring postural balance in young adults with CNLBP (ICC > 0.80 and SEM < 1.30) [45].

#### 2.5.3. Oswestry Disability Index and Roland Morris Disability Questionnaire

The ODI and RMDQ were the primary outcomes used to assess self-reports of disability caused by LBP [46,47]. The ODI is made up of 10 sections scored from 0 to 5 points, with the total score ranging from 0 to 50 points [46]. In contrast, the RMDQ consists of 24 items with scores ranging from 0 to 24 points [46]. A higher score on both questionnaires indicates a higher level of disability [46,48]. The ODI (ICC > 0.84) and RMDQ (ICC > 0.88) have been recognized as valid and reliable tools for patients suffering from chronic LBP [49,50,51,52,53] and have been validated for the Portuguese language culture [54,55].

#### 2.5.4. Tampa Scale of Kinesiophobia

The TSK is a 17-item self-report questionnaire designed to assess fear of movement, fear of physical activity, and fear avoidance beliefs in patients with chronic LBP [56,57]. Each item has a 4-point Likert scale ranging from 0 (strongly disagree) to 4 (strongly agree), with individual scores for items 4, 8, 12, and 16 reversed. The total score is the sum of all items, ranging from 17 to 68 points, with higher scores indicating a higher level of kinesiophobia [57]. TSK is a valid and reliable tool (ICC = 0.89) for treating patients with chronic LBP [58,59] and has been validated for the Portuguese language culture [60].

#### 2.5.5. Fear Avoidance Beliefs Questionnaire

The FABQ has two scales: a four-item FABQ physical activity scale (FABQ-activity) and a seven-item work scale (FABQ-work). Each item has a 6-point Likert scale, ranging from 0 (strongly disagree) to 6 (strongly agree) [61,62]. The total score is the sum of all items ranging from 0 to 24 for FABQ-activity and 0 to 42 for FABQ-work, with a higher score indicating a higher level of fear and avoidance behavior associated with beliefs about how physical activity affects LBP. The FABQ has good internal consistency (α = 0.70) and high test–retest reliability (*r* = 0.64) for acute LBP [63], and has been validated for the Portuguese language culture [60].

#### 2.5.6. Pain Catastrophizing Scale

The PCS is a 13-item scale that measures the effect of LBP on rumination, amplification of pain sensations, and feelings of helplessness to control pain [64]. Each item has a 4-point scale ranging from 0 (not at all) to 4 (all the time), on which the participant indicates their level of thoughts and feelings while they are in pain. The total score is the sum of all items on a scale of 0–52 points, with higher scores indicating a higher level of pain catastrophizing [64]. The PCS has good to excellent internal consistency (α = 0.87–0.93) [65] and high test–retest reliability (Spearman *ρ* = 0.88) [66], and has been validated for the Portuguese language culture [67].

### 2.6. Statistical Analysis

Statistical analyses were performed using IBM SPSS Statistics for Windows (version 24.0; IBM Corp., Armonk, NY, USA). The statistical analysis was conducted using the principles of intention-to-treat analysis [68]. The Shapiro–Wilk and Levene tests were used to determine the normality and homogeneity of the data. The baseline characteristics and absolute changes (∆, postminus pre) in postural balance variables were examined using an unpaired Student’s *t* test. The changes in postural balance data over time and between groups were examined using a two-way repeated measures ANOVA. The Greenhouse–Geisser method was used to correct any sphericity violations. The Bonferroni post hoc correction was used to determine the specific differences shown by ANOVA. The Wilcoxon signed-rank test was used to assess within-group changes (from pre-intervention to post-intervention) in peak pain intensity, perceived disability, and pain-related fear. The Mann–Whitney *U* test was used to evaluate the between-group changes in absolute difference (∆) in these variables. The significance level was set at *p* < 0.05. The effect size (*r*) was considered trivial (0.0–<0.1), small (0.1–<0.3), moderate (0.3–<0.5), and large (≥0.5) [69]. The data were presented as the mean (or median), standard deviation, and 95% confidence interval (95% CI).

## 3. Results

### 3.1. Baseline Characteristics and Flowchart of the Participants

The baseline characteristics of the experimental groups are shown in Table 1. Baseline physical characteristics were similar among the groups (*p* > 0.05). Fifty-nine participants were screened for eligibility, and 21 were excluded for the reasons outlined in Figure 1. The remaining 38 participants were randomly assigned to one of two groups: PIL + PBMT (*n* = 19; 12 women and seven men) and PIL + SHAM (*n* = 19; 12 women and seven men), with one participant from the PIL + PBMT group being examined by intention-to-treat due to dropping out of the study.

### 3.2. Postural Balance

**Figure 4:**
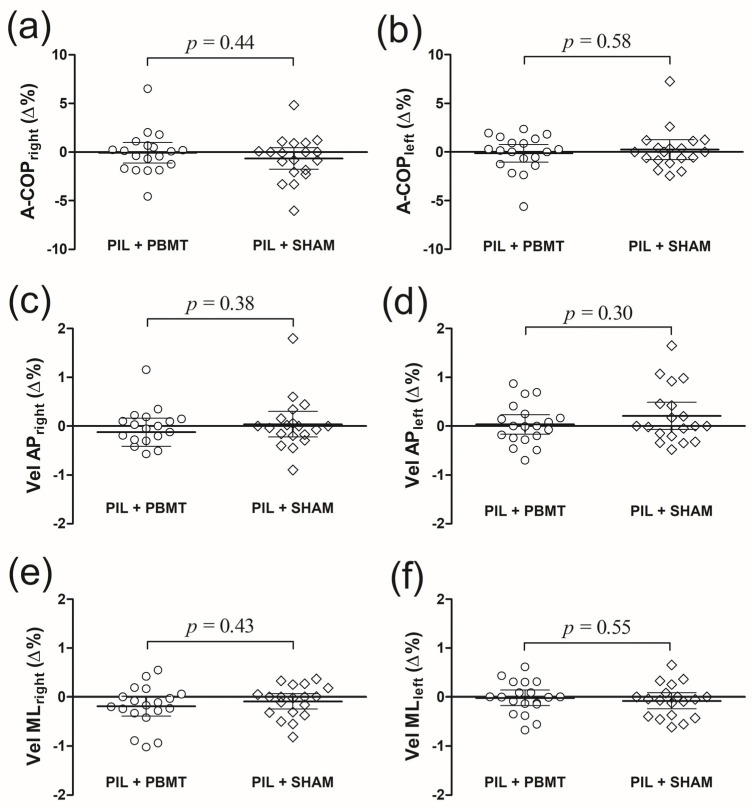
Absolute changes (∆, postminus pre) in area of the center of pressure (A-COP_right_ and A-COP_left_) (**a**,**b**), velocity anteroposterior (Vel AP_right_ and Vel AP_left_) (**c**,**d**), and velocity mediolateral (Vel ML_right_ and Vel M_Lleft_) (**e**,**f**) between PIL + PBMT and PIL + SHAM groups. The lines show the mean and 95% confidence interval, and the symbols (circle and diamond) indicate individual changes. No significant differences were found between groups (unpaired *t* test).

Figure 4 shows the absolute changes (∆) in postural balance between the PIL + PBMT and PIL + SHAM groups. A repeated–measure ANOVA revealed no statistically significant difference over time and between groups for A-COP_right_ (time, *p* = 0.32; interaction, *p* = 0.43), A-COP_left_ (time, *p* = 0.87; interaction, *p* = 0.58), Vel AP_right_ (time, *p* = 0.64; interaction, *p* = 0.38), Vel AP_left_ (time, *p* = 0.14; interaction, *p* = 0.30), Vel ML_right_ (time, *p* = 0.30; interaction, *p* = 0.43), and Vel ML_left_ (time, *p* = 0.37; interaction, *p* = 0.55). An unpaired *t* test revealed no significant difference in absolute changes (∆) between the PIL + PBMT and PIL + SHAM groups for all postural control variables (Figure 4).

### 3.3. Peak Pain Intensity, Perceived Disability, and Pain-Related Fear

Peak pain intensity, perceived disability, and pain-related fear data are shown in Table 3. A Wilcoxon signed-rank test revealed a significant reduction from pre-intervention to post-intervention in both groups for the peak pain intensity (PIL + PBMT: −30%; PIL + SHAM: −18%), perceived disability (ODI [PIL + PBMT: −26%; PIL + SHAM: −22%] and RMDQ [PIL + PBMT: −31%; PIL + SHAM: −28%]), and pain-related fear (PCS [PIL + PBMT: −38%; PIL + SHAM: −44%]), with no significant difference in the TSK and FABQ scores. The Mann–Whitney *U* test revealed similar (*p* > 0.05) absolute changes (∆) in peak pain intensity, perceived disability (ODI and RMDQ scores), and pain-related fear (PCS score) between PIL + PBMT and PIL + SHAM groups.

## 4. Discussion

This study aimed to investigate the effects of an 8-week Pilates exercise program combined with PBMT in the treatment of CNLBP. We hypothesized that the mat Pilates program combined with PBMT would be more effective than the Pilates program alone in improving postural balance and decreasing pain intensity, perceived disability, and pain-related fear of movement in patients with CNLBP. The study’s major findings were that mat Pilates alone reduced peak pain intensity, perceived disability (ODI and RMDQ scores), and PCS score, with no additional effect from PBMT on these variables. Furthermore, mat Pilates alone or in combination with PBMT did not improve postural balance (A-COP, Vel AP, and Vel ML). Thus, our findings did not support the hypothesis that Pilates + PBMT could be more effective than Pilates alone in the treatment of CNLBP.

Low back pain. Our findings revealed a similar reduction in peak pain intensity between the PIL + PBMT and PIL + SHAM groups. These findings are consistent with a recent meta-analysis study (containing 17 RCTs) that found a beneficial effect of Pilates on the pain scale (SMD = −1.31) in patients with chronic LBP [70]. However, it is important to note that only one of the 17 RCTs used mat Pilates [36], whereas the other 16 studies used various Pilates protocols alone or in combination with other treatments (i.e., standardized education program, infrared radiation, back care, tendon puncture, suspension training, a nonsteroidal anti-inflammatory drug, and massage). Understanding the specific effects of various forms of Pilates is crucial for future research and evidence-based treatment. As a result, our study adds to the current body of data by showing that mat Pilate had a positive effect on peak pain intensity in patients with CNLBP.

Furthermore, the combination of Pilates and PBMT was not more effective in reducing pain than Pilates alone. Although PBMT has been proposed to reduce levels of inflammatory mediators [23], such as prostaglandin E_2_ [24], and stimulate stem cells and progenitor cells (i.e., muscle satellite cells) associated with muscle regeneration [22], recent evidence from a meta-analytic study [25] involving 12 RCTs (*n* = 1046) does not support the use of PBMT to reduce pain and disability in people with CNLBP. Guimarães et al. [71] found that PBMT did not significantly reduce pain and disability in patients with CNLBP who had 12 treatment sessions (three times a week) for 4 weeks. In line with these findings, we showed for the first time that PBMT had no therapeutic effect on peak pain intensity in patients with CNLBP following a mat Pilates program. It is important to note that previous studies [27,72,73,74] using other types of exercise (e.g., aquatic and multimodal exercise) found that PBMT had an additional effect on pain intensity in patients with CNLBP. For example, a previous study from our laboratory found that a 4-week aquatic exercise program plus active PBMT resulted in a higher reduction in pain intensity (ES = 1.58) than exercise alone (sham PBMT; ES = 0.92) [72]. In addition, Tantawy et al. [27] found that an 8-week multicomponent exercise program combined with PBMT resulted in a higher reduction in pain intensity in patients with CNLBP than exercise alone. Therefore, the effectiveness of PBMT as an adjunctive strategy in the treatment of CNLBP may be dependent on the protocol and type of physical exercise. It is also important to mention that the lack of effectiveness of PBMT found in the present study is restricted to the parameters used. Future studies using other PBMT parameters (e.g., total dose, energy density, device power, light sources, etc.) are needed to confirm our findings.

Postural balance. Our results also indicated that mat Pilates alone (PIL + SHAM) or in combination with PBMT (PIL + PBMT) did not improve postural balance variables. In contrast to our findings, previous studies have indicated that Pilates can improve postural balance in young adults with chronic LBP [35,38,75]. It is important to note that our participants showed better postural balance at baseline than in previous studies [35,38,75], as evidenced by lower A-COP (approximately 8 cm^2^), Vel AP (approximately 2.5 cm/s), and Vel ML (approximately 3.5 cm/s). In Lopes’ study [75], for example, participants exhibited a higher baseline A-COP (approximately 11.5 cm^2^), indicating poorer postural balance, which may support the significant effect of Pilates. In fact, it is widely recognized that exercise-induced changes in proprioceptive and neuromuscular mechanisms are more prominent in individuals with more impaired systems. Therefore, the lack of effect of Pilates on postural balance in our sample is not surprising, and it indicates that the effect of Pilates on postural balance may vary depending on the patient’s level of balance impairment. In addition, PBMT did not improve postural balance in our study. To our knowledge, only one previous study [76] has investigated the effects of PBMT in combination with a physical exercise program on postural balance in adults with CNLBP. Similarly to our study, the authors found no additional effects of PBMT plus stabilization training when compared with stabilization training alone at short (3 weeks) and long term (3 months), indicating that the lack of effect of PBMT on postural balance cannot be attributed to treatment duration. In addition, a previous study in our laboratory found no effect of PBMT in combination with a 10-week resistance training program on postural control in older women, indicating that the lack of effect of PBMT on postural control is not restricted to patients with CNLBP. Taking into account that postural balance is an integral part of maintaining activities of daily living and that individuals with chronic LBP have poorer postural control than their healthy peers [10], it is suggested that more studies be performed to determine the effects of PBMT associated with exercise on postural balance in people with CNLBP, particularly the optimization of PBMT parameters.

Perceived disability and pain-related fear. The present study indicated that mat Pilates alone (PIL + SHAM) reduced perceived disability (ODI and RMDQ scores), whereas PBMT (PIL + PBMT) did not optimize these effects. The lack of an additional effect of PBMT found in our study may be attributed to its inability to reduce pain and disability in patients with CNLBP [25,71]. On the other hand, the beneficial effect of Pilates on perceived disability is not surprising given recently published data on Pilates versus no treatment [15,70] and other exercise modalities [19]. It appears that any Pilates stimulus that is sufficient in frequency (one to two sessions a week) and duration (3–9 weeks) has the potential to reduce perceived disability [19], although previous meta-analysis studies reported a low-to-moderate level of quality of evidence [15,19], indicating the need for more high-quality studies to confirm the effectiveness of Pilates on this outcome in people with CNLBP. Our findings support this notion by showing that an 8-week Pilates program (three times a week) decreased perceived disability in adults with CNLBP, with a moderate within-group effect. This beneficial effect of Pilates on perceived disability can be explained by the significant reduction in PCS score, as high catastrophizers to pain have a higher disability than low catastrophizers to pain [33], and pain catastrophizing can predict disability in patients with chronic LBP [77]. However, it is important to note that Pilates alone or in combination with PBMT did not reduce other subjective indicators of pain-related fear (FABQ-activity and TSK scores), indicating that Pilates training may be more beneficial for PCS dimensions than kinesiophobia and fear avoidance beliefs. Thus, a multifaceted approach to pain-related fear is required to determine the effectiveness of Pilates programs in patients with CLBP.

This investigation has inherent limitations. First, the intervention period of 8 weeks does not allow us to assess if the results would alter if the period were longer. Second, the findings are limited to adults and should not be generalized to other age groups. Third, we did not examine indicators of muscle activity (e.g., electromyographic signal) and biochemical markers associated with pain to draw more robust conclusions from our findings. In contrast, we should emphasize the strengths of our studies. First, this is the first study to investigate the effects of Pilates plus PBMT on adult patients with CNLBP. Second, we used gold-standard equipment to evaluate postural balance (a force platform), which improved the data’s reliability and validity. Third, Pilates sessions were progressive and individually supervised by a physiotherapist certified in Pilates. Finally, we used a double-blind approach in which the participant and the physiotherapist who administered the exercises were blinded to the PBMT treatment.

## 5. Conclusions

Our results show that mat Pilates alone can reduce peak pain intensity, perceived disability, and pain catastrophizing in adults with CNLBP, with no additional effect from PBMT on these variables. Mat Pilates alone or in combination with PBMT did not improve postural balance. Further research into other PBMT parameters (e.g., dose, timing, and irradiation source) and Pilates methods (e.g., equipment-based Pilates) is required to confirm our findings.

## Figures and Tables

**Table 1:** Baseline characteristics in the PIL + PBMT (*n* = 19) and PIL + SHAM (*n* = 19) groups.

	PIL + PBMT			PIL + SHAM			Group *p*-Value
	Women (*n* = 12)	Men (*n* = 7)	Group (*n* = 19)	Women (*n* = 12)	Men (*n* = 7)	Group (*n* = 19)	
Age (year)	47.9 (12.4)	46.7 (10.2)	47.5 (11.4)	47.9 (12.0)	44.6 (9.6)	46.7 (11.0)	0.83
Height (cm)	160.4 (7.3)	174.1 (7.1)	165.5 (9.8)	158.3 (7.9)	172.7 (4.5)	163.6 (9.8)	0.55
Weight (kg)	74.0 (12.2)	95.0 (13.0)	81.7 (16.0)	73.6 (14.6)	84.2 (12.8)	79.2 (14.1)	0.62
BMI (kg/m^2^)	28.8 (4.7)	31.3 (4.1)	29.7 (4.5)	30.6 (5.8)	28.2 (4.0)	29.7 (5.2)	0.98
Duration of LBP (year)	3.0 (0.9)	2.0 (1.0)	2.6 (1.0)	2.5 (0.8)	2.3 (1.0)	2.4 (0.8)	0.49

Note: BMI, body mass index; LBP, low back pain. Values are mean (SD, standard deviation). There were no differences between the groups at *p* < 0.05 (unpaired *t* test).

**Table 2:** Irradiation parameters in the PIL + PBMT (*n* = 19) and PIL + SHAM (*n* = 19) groups.

Parameters	Red (RED)	Infrared (IR)
Number of LEDs	132	132
Wavelength	660 nm	850 nm
Frequency (Hz)	Continuous	Continuous
Effective irradiation area (each LED)	0.5 cm^2^	0.5 cm^2^
Mean power (each LED)	8 mW	8 mW
Total power (device)	2.11 W (2112 mW)	
Effective area irradiation (device)	132 cm^2^ (0.5 cm^2^ × 264 LED)	
Power density/irradiance (device)	16 mW/cm^2^ (2112 mW/132 cm^2^)	
Treatment time	20 min (1200 s)	
Total energy/dose	2532 J (2.11 W × 1200 s)	
Energy density/fluency	19.2 J/cm^2^ (2532 J/132 cm^2^)	
Mode of application	Contact	

**Table 3:** Mean and peak low back pain, perceived disability, and pain-related fear in the PIL + PBMT (*n* = 19) and PIL + SHAM (*n* = 19) groups.

	Wilcoxon Signed Rank Test (Within-Group Change)							Mann–Whitney *U* Test (Between-Group Change)						
	*n*	Pre *Md*	Post *Md*	*Z*	*p*-Value	Effect Size *r*		∆ *Md*	*U*	*W*	*Z*	*p*-Value	**Effect Size** ***r***	
***Peak pain intensity***														
PIL + PBMT	19	5.00	3.00 *	−2.59	0.01	0.42	*Mod.*	−1.00	135.00	325.00	−1.35	0.18	0.22	*Small*
PIL + SHAM	19	5.00	3.00 *	−2.38	0.02	0.39	*Mod.*	*0.00*						
***Perceived disability***														
***ODI***														
PIL + PBMT	19	11.00	9.00 *	−2.61	0.01	0.42	*Mod.*	−4.00	151.50	341.50	−0.85	0.40	0.14	*Small*
PIL + SHAM	19	12.00	8.00 *	−2.28	0.02	0.37	*Mod.*	−3.00						
***RMDQ***														
PIL + PBMT	19	12.00	9.00 *	−2.51	0.01	0.41	*Mod.*	−2.00	166.50	356.50	−0.41	0.69	0.07	*Trivial*
PIL + SHAM	19	11.00	7.00 *	−3.12	0.00	0.50	*Mod.*	−3.00						
***Pain-related fear***														
***PCS***														
PIL + PBMT	19	29.00	19.00 *	−3.36	0.001	0.54	*Large*	−10.00	175.00	365.00	−0.16	0.88	0.03	*Trivial*
PIL + SHAM	19	32.00	14.00 *	−3.46	0.001	0.56	*Large*	−8.00						
***TSK***														
PIL + PBMT	19	37.00	36.00	−1.49	0.13	0.24	*Small*	−5.00	144.00	334.00	−1.07	0.29	0.17	*Small*
PIL + SHAM	19	39.00	36.00	−1.32	0.19	0.21	*Small*	−3.00						
***FABQ***														
PIL + PBMT	19	11.00	10.00	−1.25	0.21	0.20	*Small*	−4.00	169.50	359.5	−0.32	0.76	0.05	*Trivial*
PIL + SHAM	19	12.00	10.00	−1.52	0.13	0.25	*Small*	−2.00						

Note: FABQ, Fear Avoidance Beliefs Questionnaire; Md, median; ODI, Oswestry Disability Index; PCS, Pain Catastrophizing Scale; RMDQ, Roland Morris Disability Questionnaire; TSK, Tampa Scale of Kinesiophobia. ∆ = absolute difference (postminus pre). * *p* < 0.05 versus baseline (time effect within the group).

## Data Availability

Data is contained within the article or Supplementary Material.

## Supplementary Materials

The following supporting information can be downloaded at: https://www.mdpi.com/article/10.3390/healthcare12141416/s1, Table S1: Description of mat Pilates exercises.
