# Supplementary material for: Effects of a Mat Pilates Exercise Program Associated with Photobiomodulation Therapy in Patients with Chronic Nonspecific Low Back Pain: A Randomized, Double-Blind, Sham-Controlled Trial
Source: Healthcare (Basel). 2024 Jul 16;12(14):1416. doi: 10.3390/healthcare12141416 (PMC11276592; doi:10.3390/healthcare12141416)
Supplement: Supplementary file 1 [file healthcare-12-01416-s001.zip › healthcare-3066265-supplementary.pdf]

SUPPLEMENTARY TABLE S1.

**Supplementary Table S1**. Description of mat Pilates exercises		
**Week 1**		
**01**	Patient in supine position, hips and legs flexed, feet flat on the floor, cervical spine neutral and arms along the trunk. Patient should inhale slowly, and on exhalation perform the activation of the *power house* (activating transverse abdominis). The activation of the transversus abdominis should remain until the end of the exhalation, thus starting the cycle again.	Pre-pilates (breath awareness) – activation of the *power house*
**02**	Patient in spine position, with hip flexion (90º) and knees, arms along the trunk and neutral cervical. Patient should inhale slowly and on exhalation should be taking the arms behind the head and returning on inspiration (repeating the cycle).	Pre-pilates – mobilization of the shoulder girdle (bilateral shoulder flexion)
**03**	Patient in spine position, with flexion (90º) and knees, arms along the trunk and cervical and neutral spine. Patient should inhale and exhale slowly, and at the same time performing antero and retroversion movements of the pelvic girdle.	Pre-pilates – mobilization of the pelvic girdle (antero/retroversion)
**04**	Patient seated hip and knee flexion, feet flat on the floor and hands holding the knees and looking forward. Patients should inhale and on exhalation should be activating the power house, bringing the chin to the chest and making a C with the spine, slipping the hands and slowly lowering the trunk. At the end of the exhalation, the patient should inhale	Roll down – spine mobilization – column in "C"

	again and return doing the movement in reverse.<br>Return to the starting position.	
**05**	Patient in supine position, hips and knees flexed, feet on the floor, cervical and spine neutral and arms along the trunk. Patient should perform and inspiration and on exhalation bring one leg in hip flexion (90º) and returning on inspiration. Patient should perform this movement alternating right leg and left leg.<br>Return to the starting position.	Pre-pilates – mobilization of the pelvic girdle (hip and knee flexion)
**06**	Patient in supine position, with hip flexion (90º) and knees flexed, arms along the trunk ("floating"), chin against the chest and spine in C (table top position). Patient should inhale and exhale continuously triggering the *power house* and at the same time performing a "pumping" movement with the arms.	The hundred - "table top"
**Week 2**		
**01**	Patient in supine position, hips and legs flexed, feet flat on the floor, cervical spine neutral and arms along the trunk. Patient should inhale slowly, and on exhalation perform the activation of the *power house* (activating transverse abdominis). The activation of the transversus abdominis should remain until the end of the exhalation, thus starting the cycle again.	Pre-pilates (breath awareness) – activation of the *power house*
**02**	Patient in spine position, with hip flexion (90º) and knees, arms along the trunk and neutral cervical. Patient should inhale slowly and on exhalation should be taking the arms behind the head and returning on inspiration (repeating the cycle).	Pre-pilates - mobilization of the shoulder girdle (bilateral shoulder flexion)

**03**	Patient in spine position, with flexion (90º) and knees, arms along the trunk and cervical and neutral spine. Patient should inhale and exhale slowly, and at the same time performing antero and retroversion movements of the pelvic girdle.	Pre-pilates – mobilization of the pelvic girdle (antero/retroversion)
**04**	Patient seated hip and knee flexion, feet flat on the floor and hands holding the knees and looking forward. Patients should inhale and on exhalation should be activating the power house, bringing the chin to the chest and making a C with the spine, slipping the hands and slowly lowering the trunk. At the end of the exhalation, the patient should inhale again and return doing the movement in reverse.<br>Return to the starting position.	Roll down – spine mobilization – column in “C”
**05**	Patient in supine position, hips and knees flexed, feet on the floor, cervical and spine neutral and arms along the trunk. Patient should perform and inspiration and on exhalation bring one leg in hip flexion (90º) and returning on inspiration. Patient should perform this movement alternating right leg and left leg.<br>Return to the starting position.	Pre-pilates – mobilization of the pelvic girdle (hip and knee flexion)
**06**	Patient in supine position, with hip flexion (90º) and knees flexed, arms along the trunk (“floating”), chin against the chest and spine in C (table top position). Patient should inhale and exhale continuously triggering the *power house* and at the same time performing a “pumping” movement with the arms.	The hundred - “table top”
**07**	Patient in supine position, legs extended and joined, feet in dorsiflexion, knees locked and arms behind the head (shoulder width).	

	Patient should inhale slowly and on exhalation, should bring the arms forward and the head tilting it, starting to roll slowly (making a C with the spine and chin trying to touch the chest); removing the back from the mattress vertebra by vertebra, directing both hands to the feet.<br>Return to the starting position.	Roll up
**08**	Patient in prone position, legs extended and joined, arms extended (front) and joined, and looking down. Patient should inhale slowly and on exhalation, he should be lifting the upper limb off the floor and simultaneously the contralateral leg. The patient should be simulating a swim (alternating arms and legs).<br>Return to the starting position.	Swimming (trunk upper limb and lower limb)
**09**	Patient in supine position with on leg flexed holding one hand at the ankle and the other hand at the knee. The contralateral leg should be in approximately 15º flexion. The patient should keep the chin against the chest and the spine in a C. Patient should inhale slowly and exhalation exchange legs and hands, performing the alternating movement.<br>Return to the starting position.	One leg stretch
**10**	Patient in spine position, with hip and knee flexion, hugging both legs, chin against the chest and C spine. Patient inhales slowly, and on exhalation the patient extends both knees, stretching the legs, and both arms are directed behind the head, making a turn around the trunk, and finding both legs again embracing them. Starting the movement again.<br>Return to the starting position.	Double leg stretch
**Week 3**		

**01**	Patient in supine position, hips and legs flexed, feet flat on the floor, cervical spine neutral and arms along the trunk. Patient should inhale slowly, and on exhalation perform the activation of the *power house* (activating transverse abdominis). The activation of the transversus abdominis should remain until the end of the exhalation, thus starting the cycle again.	Pre-pilates (breath awareness) – activation of the *power house*
**02**	Patient in spine position, with hip flexion (90º) and knees, arms along the trunk and neutral cervical. Patient should inhale slowly and on exhalation should be taking the arms behind the head and returning on inspiration (repeating the cycle).	Pre-pilates - mobilization of the shoulder girdle (bilateral shoulder flexion)
**03**	Patient in spine position, with flexion (90º) and knees, arms along the trunk and cervical and neutral spine. Patient should inhale and exhale slowly, and at the same time performing antero and retroversion movements of the pelvic girdle.	Pre-pilates – mobilization of the pelvic girdle (antero/retroversion)
**04**	Patient seated hip and knee flexion, feet flat on the floor and hands holding the knees and looking forward. Patients should inhale and on exhalation should be activating the power house, bringing the chin to the chest and making a C with the spine, slipping the hands and slowly lowering the trunk. At the end of the exhalation, the patient should inhale again and return doing the movement in reverse.<br>Return to the starting position.	Roll down – spine mobilization – column in "C"
**05**	Patient in supine position, hips and knees flexed, feet on the floor, cervical and spine neutral and arms along the trunk. Patient should perform and inspiration and on exhalation bring one leg in hip	Pre-pilates – mobilization of the pelvic girdle (hip and knee flexion)

	flexion (90º) and returning on inspiration. Patient should perform this movement alternating right leg and left leg.<br>Return to the starting position.	
**06**	Patient in supine position, with hip flexion (90º) and knees extended, arms along the trunk ("floating"), chin against the chest and spine in C. Patient should inhale and exhale continuously triggering the *power house* and at the same time performing a "pumping" movement with the arms.	The hundred (hip flexion at 90º)
**07**	Patient in supine position, legs extended and joined, feet in dorsiflexion, knees locked and arms behind the head (shoulder width). Patient should inhale slowly and on exhalation, should bring the arms forward and the head tilting it, starting to roll slowly (making a C with the spine and chin trying to touch the chest); removing the back from the mattress vertebra by vertebra, directing both hands to the feet.<br>Return to the starting position.	Roll up
**08**	Patient in prone position, legs extended and joined, arms extended (front) and joined, and looking down. Patient should inhale slowly and on exhalation, he should be lifting the upper limb off the floor and simultaneously the contralateral leg. The patient should be simulating a swim (alternating arms and legs).<br>Return to the starting position.	Swimming
**09**	Patient in supine position with on leg flexd holding one hand at the ankle and the other hand at the knee. The contralateral leg should be in approximately 15º flexion. The patient should keep the chin against	One leg stretch

	the chest and the spine in a C. Patient should inhale slowly and exhalation exchange legs and hands, performing the alternating movement.<br>Return to the starting position.	
**10**	Patient in spine position, with hip and knee flexion, hugging both legs, chin against the chest and C spine. Patient inhales slowly, and on exhalation the patient extends both knees, stretching the legs, and both arms are directed behind the head, making a turn around the trunk, and finding both legs again embracing them. Starting the movement again.<br>Return to the starting position.	Double leg stretch
**11**	Patient seated with legs apart (feet in dorsiflexion), arms extended (forward) and neck neutral (looking forward). Patient should inhale slowly and on exhalation, should be bring his arms forward (trying to reach feet), making a C with the spine.<br>Return to the starting position.	Spine stretch
**12**	Patient seated, with legs flexed (one leg in front and the other behind). Arms open (90º), palms facing forward. Patient should inhale slowly and exhalation, he should be tilting the trunk sideways (of the leg that is in front), and contralateral arm should be passing over the head and the other arm (relaxed over the leg). The same movement will be performed for the other side.<br>Return to the starting position.	Mermaid
**13**	Patient seated, legs apart, feet in dorsiflexion, arms (shoulder height) abducted (joining the scapulae and pressing them down), and head (looking forward). Patient should inhale slowly and on exhalation, he should be turning the trunk to right	The saw

	side, and the left hands should be crossing the right foot (lateral malleolus), stretching the body forward (as much as possible) performing three successive "saw" slides. Patient should be returning and performing the movement to the other side.<br>Return to the starting position.	
**14**	Patient in prone position, forearms resting on thr floor (90º angle), and hands resting on the floor, chest elevated, with scapulae arranged, cervical spine neutral, and legs extended and joined. Patient should flex one the knees (90º), kicking with the tip of the foot, twice (trying to touch gluteus), alternating legs.<br>Return to the starting position.	The one leg kick
**Weeks 4-8**		
**01**	Patient in supine position, hips and legs flexed, feet flat on the floor, cervical spine neutral and arms along the trunk. Patient should inhale slowly, and on exhalation perform the activation of the *power house* (activating transverse abdominis). The activation of the transversus abdominis should remain until the end of the exhalation, thus starting the cycle again.	Pre-pilates (breath awareness) – activation of the *power house*
**02**	Patient in spine position, with hip flexion (90º) and knees, arms along the trunk and neutral cervical. Patient should inhale slowly and on exhalation should be taking the arms behind the head and returning on inspiration (repeating the cycle).	Pre-pilates – mobilization of the shoulder girdle (bilateral shoulder flexion)
**03**	Patient in spine position, with flexion (90º) and knees, arms along the trunk and cervical and neutral spine. Patient should inhale and exhale slowly, and at the same time	Pre-pilates – mobilization of the pelvic girdle (antero/retroversion)

	performing antero and retroversion movements of the pelvic girdle.	
**04**	Patient seated hip and knee flexion, feet flat on the floor and hands holding the knees and looking forward. Patients should inhale and on exhalation should be activating the power house, bringing the chin to the chest and making a C with the spine, slipping the hands and slowly lowering the trunk. At the end of the exhalation, the patient should inhale again and return doing the movement in reverse. Return to the starting position.	Roll down – spine mobilization – column in “C”
**05**	Patient in supine position, hips and knees flexed, feet on the floor, cervical and spine neutral and arms along the trunk. Patient should perform and inspiration and on exhalation bring one leg in hip flexion (90º) and returning on inspiration. Patient should perform this movement alternating right leg and left leg. Return to the starting position.	Pre-pilates – mobilization of the pelvic girdle (hip and knee flexion)
**06**	Patient in spine position, with hip flexion (15º) and legs extended (close to the floor), arms along the trunk (“floating”), chin against the chest and spine in C. Patient should inhale and exhale continuously triggering the power house and at the same time performing a “pumping” movement with the arms.	The hundred (legs at 15º)
**07**	Patient in supine position, legs extended and joined, feet in dorsiflexion, knees locked and arms behind the head (shoulder width). Patient should inhale slowly and on exhalation, should bring the arms forward and the head tilting it, starting to roll slowly (making a C with the spine and chin trying to	Roll up

	touch the chest); removing the back from the mattress vertebra by vertebra, directing both hands to the feet.<br>Return to the starting position.	
**08**	Patient in prone position, legs extended and joined, arms extended (front) and joined, and looking down. Patient should inhale slowly and on exhalation, he should be lifting the upper limb off the floor and simultaneously the contralateral leg. The patient should be simulating a swim (alternating arms and legs).<br>Return to the starting position.	Swimming
**09**	Patient in supine position with on leg flexed holding one hand at the ankle and the other hand at the knee. The contralateral leg should be in approximately 15º flexion. The patient should keep the chin against the chest and the spine in a C. Patient should inhale slowly and exhalation exchange legs and hands, performing the alternating movement.<br>Return to the starting position.	One leg stretch
**10**	Patient in spine position, with hip and knee flexion, hugging both legs, chin against the chest and C spine. Patient inhales slowly, and on exhalation the patient extends both knees, stretching the legs, and both arms are directed behind the head, making a turn around the trunk, and finding both legs again embracing them. Starting the movement again.<br>Return to the starting position.	Double leg stretch
**11**	Patient seated with legs apart (feet in dorsiflexion), arms extended (forward) and neck neutral (looking forward). Patient should inhale slowly and on exhalation, should be bring his arms forward (trying to	Spine stretch

	reach feet), making a C with the spine.<br>Return to the starting position.	
**12**	Patient seated, with legs flexed (one leg in front and the other behind). Arms open (90º), palms facing forward. Patient should inhale slowly and exhalation, he should be tilting the trunk sideways (of the leg that is in front), and contralateral arm should be passing over the head and the other arm (relaxed over the leg). The same movement will be performed for the other side.<br>Return to the starting position.	Mermaid
**13**	Patient seated, legs apart, feet in dorsiflexion, arms (shoulder heigth) abducted (joining the scapulae and pressing them down), and head (looking forward). Patient should inhale slowly and on exhalation, he should be turning the trunk to rigth side, and the left hands should be crossing the right foot (lateral malleolus), stretching the body forward (as much as possible) performing three successive "saw" slides. Patient should be returning and performing the movement to the other side.<br>Return to the starting position.	The saw
**14**	Patient in prone position, forearms resting on the floor (90º angle), and hands resting on the floor, chest elevated, with scapulae arranged, cervical spine neutral, and legs extended and joined. Patient should flex one the knees (90º), kicking with the tip of the foot, twice (trying to touch gluteus), alternating legs.<br>Return to the starting position.	The one leg kick
**15**	Patient sits with hips and knees flexed, with hands held above knees, and toes resting on floor, and chin resting against chest. Patient should	Rolling like a ball

	inhale slowly, and on exhalation soul roll backwards (spine should be in C position, and chin resting against chest).<br>Return to the starting position.	
**16**	Patient in supine position, hips and knees flexed with feet flat on the floor, arms along the trunk (hands facing the mat) and cervical neutral. Patient should inhale slowly and on exhalation, should be lifting the pelvis (removing vertebra by vertebra), up to the height of the scapulae (arms and cervical moving). Return to the starting position.	The shoulder bridge
